# Unveiling the Unexpected: Lung Adenocarcinoma Presenting With Penile Mondor’s Disease and Multiple Thrombotic Complications

**DOI:** 10.7759/cureus.72610

**Published:** 2024-10-29

**Authors:** Mizuki Muranaka, Kazunori Tobino

**Affiliations:** 1 Respiratory Medicine, Iizuka Hospital, Iizuka, JPN

**Keywords:** cancer-associated thrombosis, lung adenocarcinoma, nonbacterial thrombotic endocarditis, penile mondor's disease, trousseau's syndrome

## Abstract

Penile Mondor's disease (PMD) is a rare condition characterized by thrombophlebitis of the superficial dorsal vein of the penis. While typically considered a benign and self-limiting condition, we present an unprecedented case where PMD was the initial presenting symptom of lung adenocarcinoma. This case report describes a 46-year-old Japanese man who presented with PMD and was subsequently diagnosed with stage IVA lung adenocarcinoma. The patient's clinical course was further complicated by other cancer-associated thrombophilic conditions, including deep vein thrombosis (DVT) and nonbacterial thrombotic endocarditis (NBTE). To our knowledge, this is the first reported case in the literature where PMD has been observed as an initial manifestation of lung cancer. The patient was treated with targeted therapy for lung cancer and anticoagulation for thrombotic complications. This case highlights the importance of considering underlying malignancies in patients presenting with unusual thrombotic events, even in anatomical locations not typically associated with cancer-related thrombosis. It also underscores the diverse and sometimes unexpected manifestations of cancer-associated thrombosis, challenging clinicians to maintain a high index of suspicion for underlying malignancies in patients presenting with atypical thrombotic events.

## Introduction

Mondor's disease of the penis, also known as penile Mondor's disease (PMD), is a rare condition characterized by thrombophlebitis of the superficial dorsal vein of the penis or its branches. This condition typically presents as a palpable, cord-like induration on the dorsal or dorsolateral aspect of the penis and primarily affects sexually active men, with the peak incidence between 20 and 40 years of age [1,2]. While the exact etiology remains unknown, PMD is often associated with local factors such as trauma, prolonged or vigorous sexual activity, sexual abstinence followed by intense activity, use of vacuum devices, and local infections [1-3]. Generally considered a benign and self-limiting condition, PMD typically resolves within four to eight weeks without specific treatment. Management is usually conservative, including nonsteroidal anti-inflammatory drugs (NSAIDs) for pain relief, local application of heat, and temporary sexual abstinence.

While Mondor's disease in other anatomical locations, particularly the thoracoabdominal region, has been associated with underlying malignancies in some cases, the literature on PMD has not traditionally linked it to systemic diseases or distant malignancies [1-3]. The association between PMD and underlying malignancies, particularly lung cancer, is extremely rare and poorly understood, with no direct evidence linking the two conditions in the current literature.

Lung cancer, on the other hand, is well-known to be associated with various paraneoplastic syndromes and thromboembolic events, collectively known as cancer-associated thrombosis [4, 5]. These thrombotic events typically manifest in more common forms, such as deep vein thrombosis (DVT) or pulmonary embolism [6]. However, the full spectrum of cancer-associated thrombosis continues to expand, challenging clinicians to maintain a high index of suspicion for underlying malignancies in patients presenting with unusual thrombotic events [7].

Here, we present a unique and unprecedented case of lung adenocarcinoma, where PMD was the initial presenting symptom. To the best of our knowledge, this is the first reported case in the literature where PMD has been observed as an initial manifestation of lung cancer. This case was further complicated by other cancer-related thrombophilic conditions, including DVT and nonbacterial thrombotic endocarditis (NBTE), illustrating the diverse and sometimes unexpected manifestations of cancer-associated thrombosis [8].

## Case presentation

A 46-year-old Japanese man with a four-week history of cough was referred to our hospital for further investigation of an anomaly detected on chest computed tomography (CT). He also complained of dorsal induration of the penis with pain. He had a 20-pack-year smoking history and drank alcoholic beverages occasionally. He denied trauma, vigorous sexual activity, dysuria, and hematuria.

**Figure 1:**
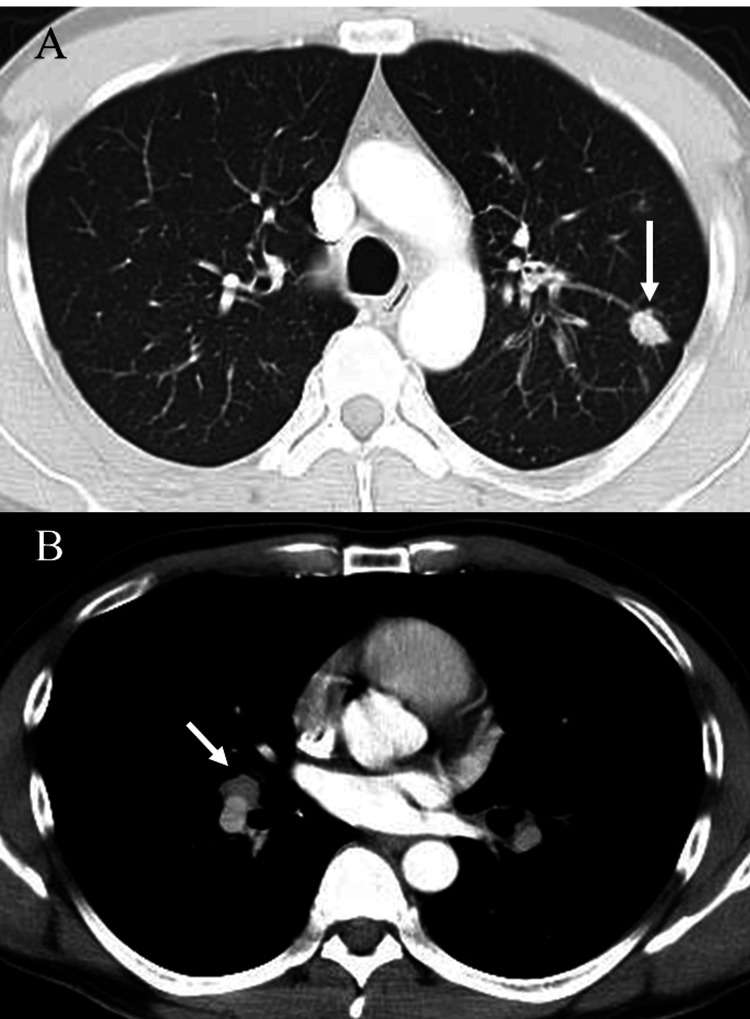
The CT scans obtained at the previous hospital Initial chest CT showed a solitary nodule in the left upper lung lobe (A, arrow) and bilateral hilar and mediastinal lymphadenopathy (B, arrow).

His initial vital signs were unremarkable. Physical examinations revealed a palpable, thick cord-like lesion on the dorsal aspect of his penis. The skin was intact. There was no inguinal lymphadenopathy. A cardiovascular examination revealed normal findings. With the exception of the patient’s D-dimer level (14.4 μg/ml; normal, < 1.0 μg/ml), his laboratory test values were unremarkable. The CT scans obtained at the previous hospital showed a solitary nodule in the left upper lung lobe (Figure 1A) and bilateral hilar and mediastinal lymphadenopathy (Figure 1B), as well as DVT in the right common femoral vein.

**Figure 2:**
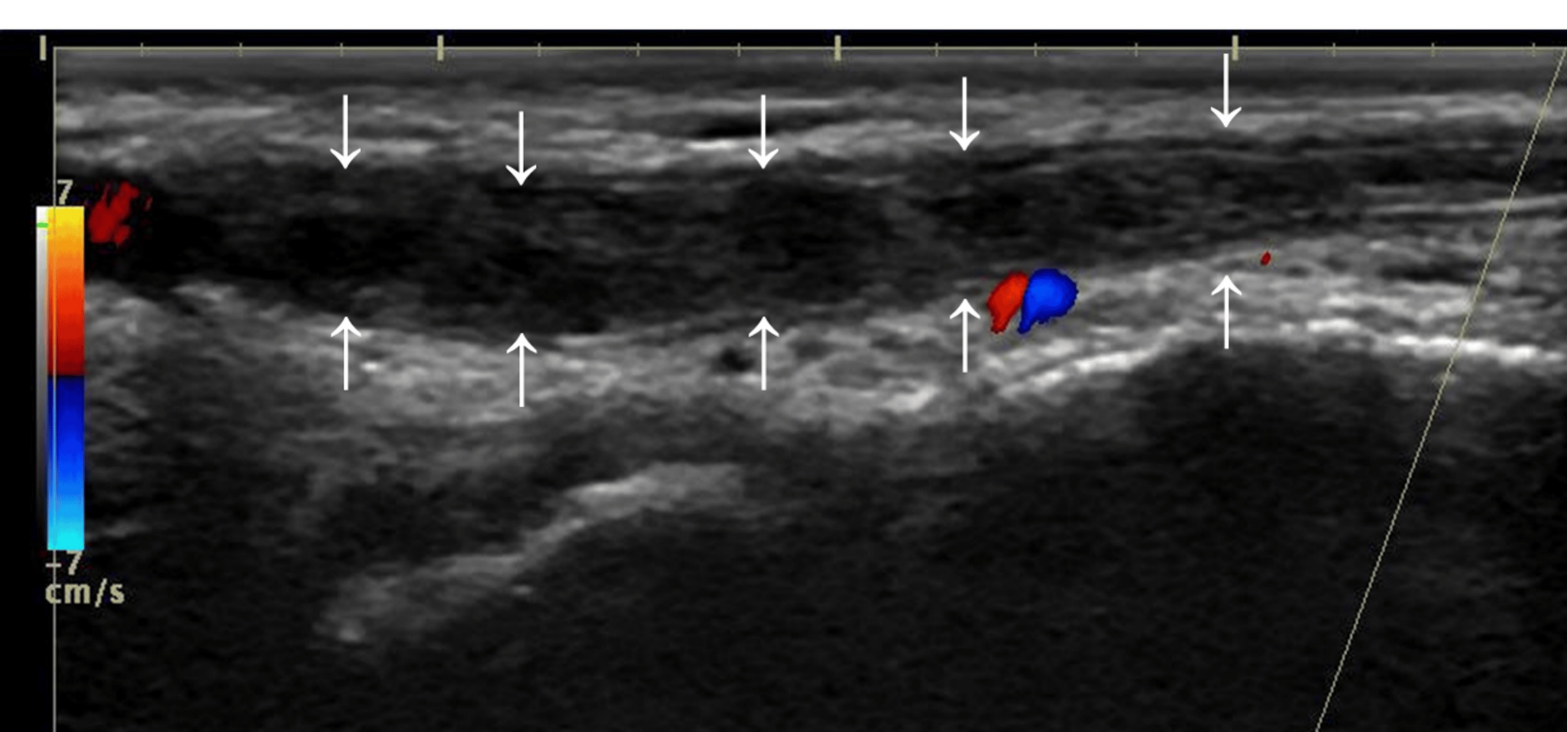
Dorsal penile vein thrombosis (arrows) detected by color Doppler ultrasonography

Pulmonary embolism (PE) was not detected. Color Doppler ultrasonography revealed thrombosis of the dorsal penile vein (Figure 2).

**Figure 3:**
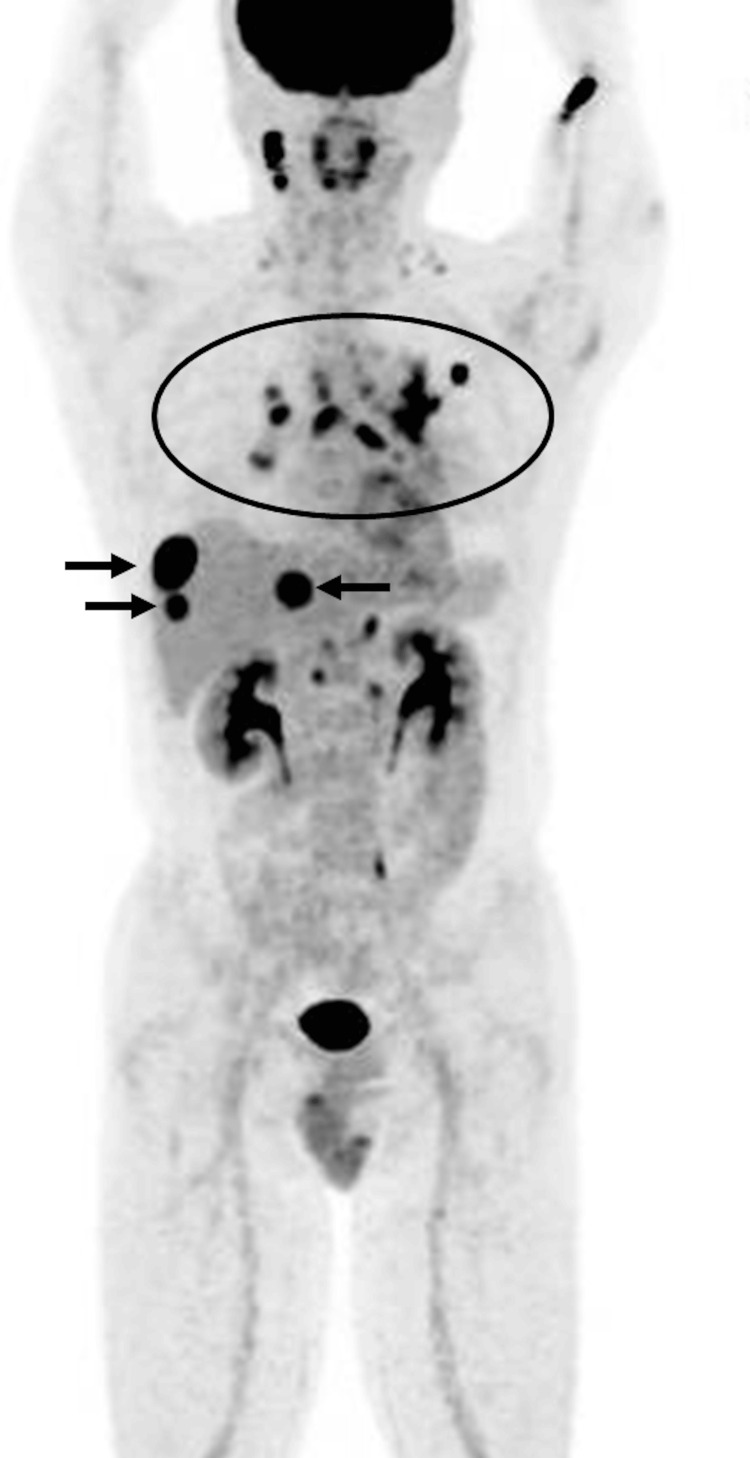
The FDG-PET showed metastases to mediastinal and hilar lymph nodes (circle), and liver (arrows). FDG-PET: 18F-fluorodeoxyglucose-positron emission tomography

A transbronchial biopsy (TBB) of the left lung nodule revealed adenocarcinoma. Brain MRI and 18F-fluorodeoxyglucose (FDG)-positron emission tomography (PET) showed metastasis to the mediastinal and hilar lymph nodes and liver (Figure 3).

Based on these results, the patient was diagnosed with lung adenocarcinoma (cT2aN3M1b, stage IVA) complicated by DVT and PMD. A molecular analysis revealed an EGFR exon 19 deletion, and afatinib therapy was started. At the same time, edoxaban was initiated for the treatment of DVT.

**Figure 4:**
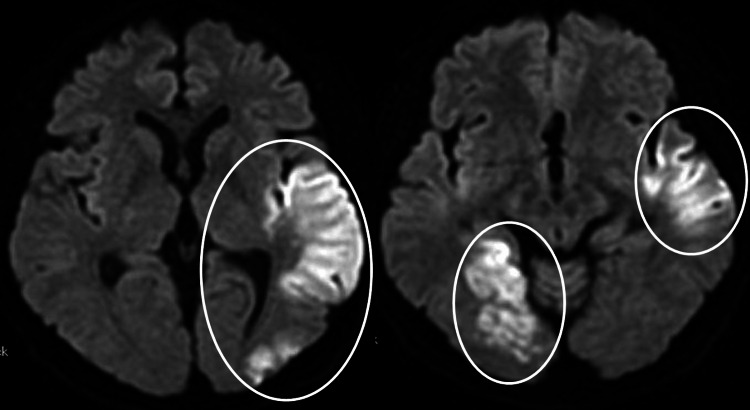
Brain MRI revealed multiple acute brain infarcts (circle).

His lung cancer regressed but subsequently progressed after ten months of stable disease. Afatinib therapy was stopped, and treatment with cisplatin and pemetrexed was initiated. One month after changing the therapy, dysarthria and visual field disturbance suddenly occurred, and a brain MRI revealed multiple acute brain infarcts (Figure 4).

**Figure 5:**
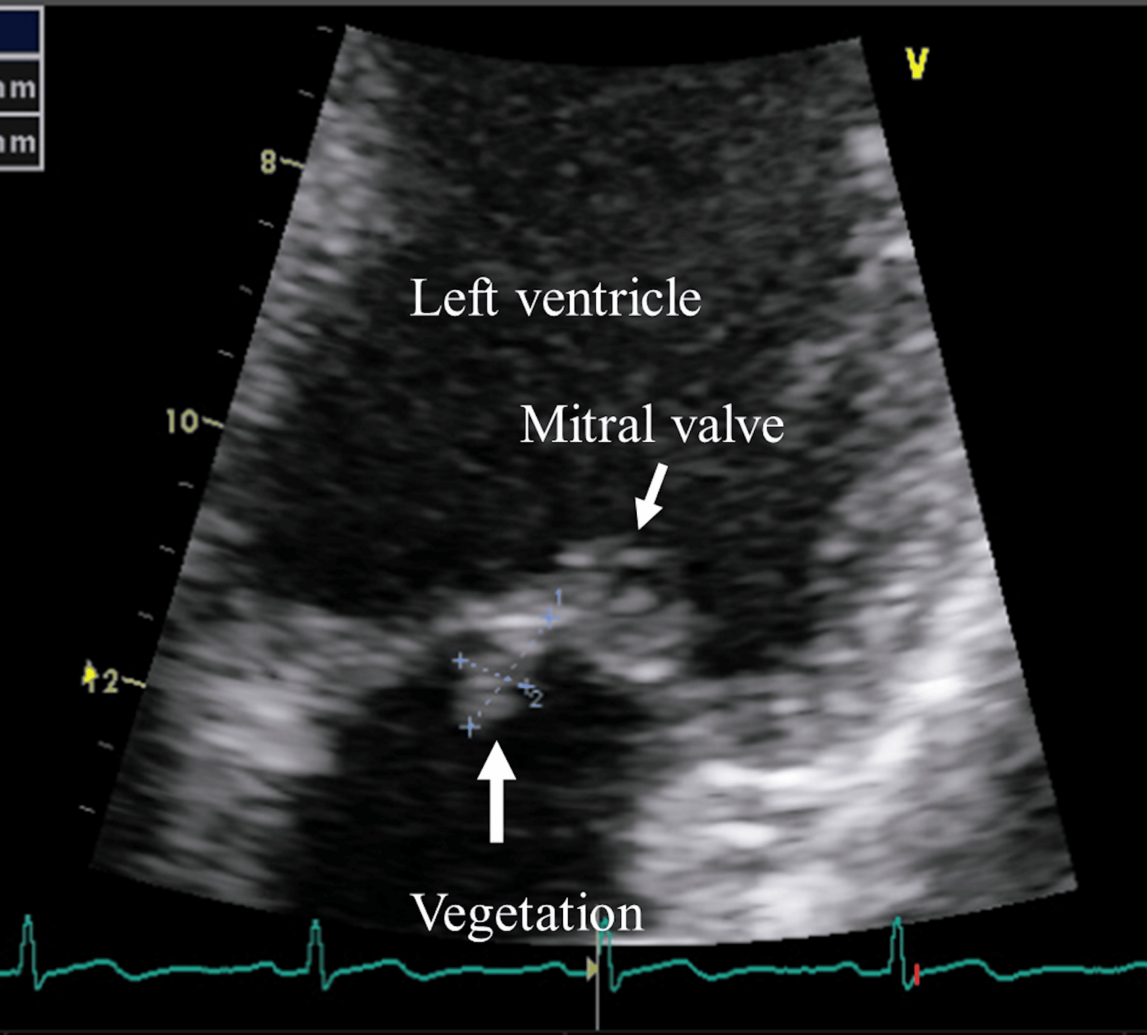
Mitral valve vegetation identified by transthoracic echocardiography A 9.9/5.2 mm nodular, hyperechogenic vegetation attached to the anterior mitral leaflet was revealed.

A cardiovascular examination revealed 2/6 early systolic murmurs at the base of the heart. He underwent transthoracic echocardiography (TTE), which showed a 9.9/5.2 mm nodular, hyperechogenic vegetation attached to the anterior mitral leaflet (Figure 5).

There were no left ventricular or atrial thrombi. An enhanced whole-body CT scan showed no signs of thrombosis or embolism in his arteries and veins, including the pulmonary vein. Repeated blood cultures did not show any microorganisms, and there were no clinical manifestations suggestive of infective endocarditis. Thus, he was diagnosed with NBTE associated with lung adenocarcinoma (i.e., Trousseau’s syndrome). Edoxaban was stopped, and heparin therapy was immediately started. His cerebral infarction did not recur after the initiation of heparin, and his condition stabilized. After 35 days of heparin therapy, the disappearance of vegetation on the anterior mitral leaflet was confirmed by TTE. His performance status improved after rehabilitation, and anticancer treatment with carboplatin and pemetrexed was started. Heparin treatment also continued. After four cycles of treatment, his lung cancer shrank. There has been no recurrence of embolism, including stroke.

## Discussion

This case report presents a unique instance of PMD as the initial presenting symptom of lung adenocarcinoma, further complicated by other cancer-associated thrombophilic conditions, including DVT and NBTE. To the best of our knowledge, this is the first reported case of PMD as an initial manifestation of lung cancer, highlighting the diverse and sometimes unexpected presentations of cancer-associated thrombosis.

Penile Mondor's disease, characterized by thrombophlebitis of the superficial dorsal vein of the penis or its branches, is a rare condition that typically presents as a palpable, cord-like induration on the dorsal or dorsolateral aspect of the penis. It primarily affects sexually active men between 20 and 40 years of age [1,2]. Previously reported risk factors for PMD include intense sexual activity, use of vacuum erection devices, trauma, prolonged erection or sitting position, urogenital infections, prostate biopsy, and hematological disorders [1-3]. However, our case is unprecedented in its association with lung cancer as an initial presentation. This case suggests that PMD may not always be a localized issue but could potentially be a sign of underlying systemic disease, particularly malignancy.

Furthermore, our patient also presented with other cancer-associated thrombophilic conditions, notably DVT and NBTE. Nonbacterial thrombotic endocarditis is a condition characterized by vegetation on cardiac valves without inflammation or bacterial infection. First described by Ziegler in 1888, the term "nonbacterial thrombotic endocarditis" was coined by Gross and Friedberg in 1936 [9,10]. Nonbacterial thrombotic endocarditis occurs in various diseases, especially malignancies [9,10]. Edoute et al. reported that NBTE was detected in 19% of 200 non-selected solid tumor patients in a retrospective study [11]. Approximately 50% of NBTE cases are reported to be associated with advanced lung and ovarian cancer [12-14].

To the best of our knowledge, only 12 cases of NBTE in patients with lung cancer have been reported in the relevant English literature [14-16]. Tamura et al. reviewed 142 autopsied patients with lung cancer and found NBTE in 11 (7.7%) patients. In their patients, NBTE was found in 13% (8/62 cases) of patients with adenocarcinoma and 8.6% (3/35 cases) of patients with squamous cell carcinoma [16]. Nonbacterial thrombotic endocarditis has also been reported in patients with tuberculosis, acquired immune deficiency syndrome [17], connective tissue disease (i.e., systemic lupus erythematosus (SLE) patients possessing antiphospholipid antibodies), indwelling pulmonary catheter, central venous catheter [9], snake bite, and overdose, and may occur as a late effect of radiation therapy.

The pathogenesis of NBTE is not fully understood, but several contributing factors have been identified. The most common factor is endothelial damage followed by contact between circulating platelets and subendothelial connective tissue. Other factors include carcinomatosis, immune complexes, hypercoagulability, and hypoxia [18]. In cancer patients, the development of NBTE is thought to involve procoagulant secretion by mucin-producing adenocarcinoma, platelet activation by mucin, and vascular endothelial damage and tissue factor activation by inflammatory cytokines (IL-1, IL-6, tumor necrosis factor) released from tumor-activated monocytes/macrophages. The clinical triad of NBTE proposed by McKay and Wahler includes cardiac murmur, multiple systemic emboli, and a history of malignancy [19]. Other clues for the diagnosis of NBTE include negative blood cultures, no therapeutic effect on infective endocarditis (IE), and unexplained cerebral embolism. Our patient had the full clinical characteristics of NBTE and neurological symptoms most frequently triggered the diagnosis. The neurologic symptoms of NBTE can vary; they include localized, diffuse, or mixed patterns [14,20]. Cerebral infarction in NBTE patients usually occurs as multiple lesions (especially on the middle cerebral artery), while that in infective endocarditis patients usually occurs as a single lesion [14,20]. The incidence of cerebral infarction gradually increases between three and 12 months after the diagnosis of cancer (5.1% at three months and 8.1% at 12 months) [21].

Regarding treatment, heparin is preferred over warfarin for cancer-associated thrombosis, including NBTE [12,13,15,22,23]. In NBTE patients, cytokines (IL-1, IL-6), the cyclooxygenase 2 gene, type 1 plasminogen activator inhibitor, cysteine protease, and tissue factor are activated, and the effects of warfarin are inhibited [12,13]. In our patient, heparin administration led to the disappearance of vegetation on the anterior mitral leaflet, as confirmed by TTE. Subsequently, anti-cancer treatment with carboplatin and pemetrexed was initiated alongside heparin. After four cycles of treatment, the lung cancer showed regression, and there has been no recurrence of embolism, including stroke.

## Conclusions

To the best of our knowledge, this case report is the first to describe PMD as an initial presenting symptom of lung adenocarcinoma. It emphasizes the importance of recognizing cancer-associated thrombophilic conditions (i.e., NBTE, PMD, and DVT) as potential indicators of lung cancer. It also highlights the utility of heparin in managing these conditions. When encountering cases of unexplained PMD or other atypical thromboses, clinicians should consider the possibility of underlying malignancy, particularly lung cancer. This case expands our understanding of the potential manifestations of cancer-associated thrombosis and underscores the need for a comprehensive approach to diagnosis and management in such cases.
